# Neohesperidin Protects Angiotensin II-Induced Hypertension and Vascular Remodeling

**DOI:** 10.3389/fphar.2022.890202

**Published:** 2022-05-23

**Authors:** Jingsi Zhang, Yuanshu Hui, Fengyi Liu, Qian Yang, Yi Lu, Yeting Chang, Qinlong Liu, Yanchun Ding

**Affiliations:** ^1^ Department of Cardiology II, The Second Hospital of Dalian Medical University, Dalian, China; ^2^ Department of Heart Function Examination, The Second Hospital of Dalian Medical University, Dalian, China; ^3^ Department of Hepatobiliary Pancreatic Surgery II, The Second Hospital of Dalian Medical University, Dalian, China

**Keywords:** hypertension, Nehesperidin, ROS, vascular remodeling, migration

## Abstract

Vascular remodeling due to hypertension is one of the major health challenges facing countries around the world. Neohesperidin, a flavonoid glycoside found in citrus fruits, is an antioxidant. Neohesperidin has been studied for a variety of diseases in addition to hypertension. In this study, angiotensin II was used to induce hypertension in mice (490 ng/kg/min, 14 days). We used H&E, Masson, immunofluorescence, dihydroethidine and qPCR to evaluate the effect of Nehesperidin (50 mg/kg/day, 16 days) on pathological hypertension in mice. Estimating the effect of Nehesperidin on human umbilical vein endothelial cells and vascular smooth muscle cells stimulated by angiotensin II. We found that neohesperidin inhibited angiotensin II-induced hypertension in mice. Neohesperidin reduced angiotensin II-induced vascular hypertrophy, fibrosis, inflammation and oxidative stress *in vivo*. Neohesperidin inhibited angiotensin II-induced ROS and DNA damage in human umbilical vein endothelial cells. Neohesperidin inhibited angiotensin II-induced migration of vascular smooth muscle cells. The results showed that Nehesperidin acts as an antioxidant and could significantly inhibit angiotensin II induced hypertension and vascular remodeling *in vitro* and *in vivo*.

## Introduction

With the acceleration of the aging process of human society, chronic diseases have become a major global public health problem. Hypertension is one of the chronic diseases with high morbidity. It is the main cause of structural and functional damage to the heart, blood vessels, brain, and kidneys. It is also the most important risk factor for cardiovascular disease. (Prieto et al., 2021). Hypertension is a multifactorial disease, which is the result of the dynamic interaction among genetic, physiological and environmental factors. The pathophysiology of hypertension involves multiple mechanisms, such as the up-regulation of the renin-angiotensin aldosterone system, the activation of the sympathetic nervous system, the disturbanceof G-protein-coupled receptor signals, and vascular inflammation (Oparil et al., 2018; Hegde and Aeddula, 2021).

In 1989, Baumbach and Heistad first proposed the concept of “vascular remodeling” in hypertension (Baumbach and Heistad, 1989). Hypertensive vascular remodeling is mainly manifested as vascular structure and function changes such as thickening of vessel wall, increasing ratio of wall thickness to lumen diameter, and decreasing number of small arteries (Schiffrin, 2012). At present, it is believed that intimal injury (endothelial dysfunction), medial thickening and adventitial matrix component rearrangement caused by hypertension are important factors in the occurrence of vascular remodeling. (Yuan et al., 2015; Chen et al., 2020; Okada et al., 2020).

Vascular endothelial cells (VECs) are a single layer of epithelial cells distributed in the innermost layer of blood vessels. When the blood pressure rises, the shear stress on the blood vessel wall will directly affect the VECs, causing mechanical damage, leading to endothelial cell dysfunction, even necrosis and shedding (Evans et al., 2021). Likewise, endothelial damage can exacerbate elevated blood pressure. VECs are the largest secretory organ in the human body and regulate the physiological functions of blood vessels through autocrine, paracrine and endocrine pathways. Vascular endothelium regulates blood pressure mainly by regulating the secretion of vasoactive factors. Endothelial cells continuously synthesize and secrete a variety of active factors. Ang II is the main active substance of the renin-angiotensin system. It plays a key role not only in regulating systemic arterial blood pressure, but also in cardiovascular function (Alghanem et al., 2021; Shao et al., 2021). Vascular smooth muscle cells (VSMCs) are mainly involved in contraction, synthesis and secretion of extracellular matrix (ECM).

The stimulation of pathological factors such as hyperlipidemia, hyperglycemia, reactive oxygen species (ROS) and high blood flow shear stress will result in vascular dysfunction (Bai et al., 2019; Chen et al., 2021). The cytotoxic process caused by ROS is so called oxidative stress. In the cardiovascular system, ROS plays an important role in the control of endothelial function and vascular tone, and plays a pathophysiological role in inflammation, hypertrophy, proliferation, apoptosis, migration, fibrosis, and angiogenesis. In the vascular wall, ROS is involved in the regulation of endothelial-dependent function, proliferation and apoptosis of vascular smooth muscle cells and endothelial cells, and remodeling of the vessel wall (Zhu et al., 2015; Cheng et al., 2016). ROS can inactivate the vasodilator nitric oxide (NO), thereby damaging vasodilator function, causing endothelial dysfunction, promoting changes in vascular tone, increasing resistance, and leading to vascular remodeling and hypertension.

Neohesperidin is a flavonoid compound and is abundant in cucurbitaceaeplaying an antioxidant and anti-inflammatory role (Zhao et al., 2019; Li et al., 2021). We have reported that Neohesperidin could inhibit cardiac remodeling induced by Ang II *in vivo* and *in vitro* (Zhang et al., 2020). The aim of this study was to investigate whether Neohesperidin could protect angiotensin II-inducedsmooth muscle thickening and endothelial injury by inhibiting oxidative stress, inflammation and fibrosis, thereby inhibiting vascular remodeling.

## Materials and Methods

### Animals and Treatment

Male C57BL/6 mice (24 g) aged from 8 to 10 weeks were selected. The mice were divided into Sham, Neohesperidin, Ang II, Ang II + Neohesperidin (six mice in each group). Mice were infused with Ang II (490 ng/kg/min, Aladdin, Ca) by Alzetmodel 1,002 micropump for 2 weeks (Zhang et al., 2020). A caudal vein injection of Neohesperidin (50 mg/kg/day) was performed for 16 days (Lu et al., 2020). All the procedures involved in the mouse study were approved by the Institutional Animal Care and Use Committee of Dalian Medical University.

### Blood Pressure Measurement

As described earlier, blood pressure was measured in awake mice using the tail-sleeve method. the blood pressure measured every 3 days. The ambient temperature was maintained at a warm room temperature (25–30°C) (Li et al., 2016).

### Histopathological

The Aortic vessels were fixed in 4% paraformaldehyde for 1 day, then embedded and sectioned. Sections were stained with hematoxylin-eosin (H&E), Mason trichrome staining, immunofluorescence (CD68 antibody, 1:100, Zen-bio) and Dihydroethidium (DHE). The positive regions were analyzed using ImageJ (Zhang et al., 2020).

### Real-Time PCR

Total RNA was extracted from Aortic vessel using TRIzol reagent (Sangon Biotech, B511311). The initial cDNA was synthesized using reverse transcriptase (Yeasen, 11141ES60) according to the manufacturer’s instructions. The cDNA was used for PCR amplification and glyceraldehyde 3-phosphate dehydrogenase (GAPDH) was used as an endogenous control. The Sequences was shown in Table 1.

**TABLE 1:** Primers used for quantitative real-time PCR analysis.

Gene	Forward primer (5′-3′)	Reverse primer (5′-3′)
m-IL-1β	GAA​ATG​CCA​CCT​TTT​GAC​AGT​G	TGG​ATG​CTC​TCA​TCA​GGA​CAG
m-IL-17	TCA​GCG​TGT​CCA​AAC​ACT​GAG	CGC​CAA​GGG​AGT​TAA​AGA​CTT
m-TNF-α	CAG​GCG​GTG​CCT​ATG​TCT​C	CGA​TCA​CCC​CGA​AGT​TCA​GTA​G
m-Collagen I	GCT​CCT​CTT​AGG​GGC​CAC​T	ATT​GGG​GAC​CCT​TAG​GCC​AT
m-Collagen III	CTG​TAA​CAT​GGA​AAC​TGG​GGA​AA	CCA​TAG​CTG​AAC​TGA​AAA​CCA​CC
m-NOX1	CCT​GAT​TCC​TGT​GTG​TCG​AAA	TTG​GCT​TCT​TCT​GTA​GCG​TTC
m-NOX2	AGT​GCG​TGT​TGC​TCG​ACA​A	GCG​GTG​TGC​AGT​GCT​ATC​AT
m-NOX4	TGC​CTG​CTC​ATT​TGG​CTG​T	CCG​GCA​CAT​AGG​TAA​AAG​GAT​G
m-α-SMA	CCC​AGA​CAT​CAG​GGA​GTA​ATG​G	TCT​ATC​GGA​TAC​TTC​AGC​GTC​A
m-GAPDH	AGG​TCG​GTG​TGA​ACG​GAT​TTG	GGG​GTC​GTT​GAT​GGC​AAC​A
h-NOX1	TTG​TTT​GGT​TAG​GGC​TGA​ATG​T	GCC​AAT​GTT​GAC​CCA​AGG​ATT​TT
h-NOX2	ACC​GGG​TTT​ATG​ATA​TTC​CAC​CT	GAT​TTC​GAC​AGA​CTG​GCA​AGA
h-NOX4	CAG​ATG​TTG​GGG​CTA​GGA​TTG	GAG​TGT​TCG​GCA​CAT​GGG​TA
p22phox	CCC​AGT​GGT​ACT​TTG​GTG​CC	GCG​GTC​ATG​TAC​TTC​TGT​CCC
h-GAPDH	ACA​ACT​TTG​GTA​TCG​TGG​AAG​G	GCC​ATC​ACG​CCA​CAG​TTT​C

### Vascular Relaxation Examinations

The thoracic aorta was carefully separated after mice were anesthetized and removed the outer membrane and fat with intraocular micro-forceps. 4 mm segments of the thoracic aorta were gently installed on the force sensor (Power lab, AD, Germany) in the cavity filled with Krebs-Henseleit solution (37°C, pH 7.35), which containing NaCl (118.3 mm), KCl (4.69 mm), CaCl_2_ (1.87 mm), MgSO_4_ (1.2 mm), K_2_HPO_4_ (1.03 mm), NaHCO_3_ (25 mm) and d-Glucose (11.1 mm) and bubbling with carbogen gas of 95% O_2_ and 5%CO_2_. To evaluate the relaxation ability of the aorta, we stimulated the aorta with norepinephrine (1^–6^ m × 10^–6^ m) until to a stationary phase, and then estimated the endothelium-dependent or endothelium-independent relaxation in response to increased concentrations of acetylcholine (ACh) or sodium nitroprusside (SNP) at a concentration gradient.

### Human Umbilical Vein Endothelial Cells and VSMC Culture

The HUCECs and VSMCs were cultured in DMEM medium [50 U/mL penicillin, 50 μg/ml streptomycin, 10% fetal bovine serum (FBS)]. After cells were treated with Neohesperidin was (20 μm) for 24 h, Ang II (2 μm) were added into cells for 24 h.

### Malondialdehyde, Superoxide Dismutase and Glutathione Peroxidase

The MDA and SOD kits were purchased from Solarbio, and the GSH-Px kit was purchased from Keygenbio (KGT006). After grouping treatment, the cells were lysed and determined on the enzyme plate analyzer according to the instructions.

### Migration

The VSMCs were divided into four groups and prepared into single cell suspension on serum-free DMEM medium. Add 200 μL DMEM medium containing 10% FBS into 24-well plates. 24 h later, DAPI staining of the upper chamber was taken out to observe the number of VSMC migration.

### Wound-Healing Assay

The VSMCs were inoculated on a 24-well culture plate and divided into four groups. Cells were cultured in a single layer with a “one” shape scratch along the bottom of the culture plate using the pipette gun tip as the 0 h time point. Photos were taken under an inverted microscope 24 h after the scratch. ImageJ software was used to measure the migration distance of cells to the injury area.

### 2′,7′-Dichlorofluorescin Diacetate

The fluorescent probe DCFH-DA (solarbio, China) were used to determine cellular reactive oxygen species (ROS) levels. The cells were incubated with DCFH-DA (10 mmol/L, 37°C, 20 min) in the dark. Use a fluorescent microplate (488 nm).

### Western Blot

After adding RIPA (Life-Lab, AP01L014), the vascular tissue was disrupted by sonication, and the supernatant was collected by centrifugation. After protein quantification by BCA regent ((Life-Lab, AP12L025), separated by SDS-PAGE, transferred to PVDF membrane, blocked, and primary antibody overnight (The CaNA, p-Erk1/2 and Erk1/2 were purchased from CST. The Tgf-β, Smad2/3, p-Smad2/3, p-p65, p65, NOX2, and NOX4 were purchased from Arigo. The SOD1, Catalase and GPX4 were purchased from ABclonal). After washing three times with TBST, the secondary antibody (Sino Biological Inc. 1:2000). was incubated for 1 h, washed four times with TBST, and ECL luminescence was developed.

### Statistical Analysis

All data were analyzed using GraphPad 8.0 software. Data were compared using one-way ANOVA, independent t-test, or chi-square test. * <0.05, ** <0.01, *** <0.001 was statistically significant compared with Saline, # <0.05, ## <0.01, ### <0.001 was statistically significant compared with Ang II.

## Results

### Neohesperidin Ameliorates Ang II Induced Hypertension in Mice

**FIGURE 1:**
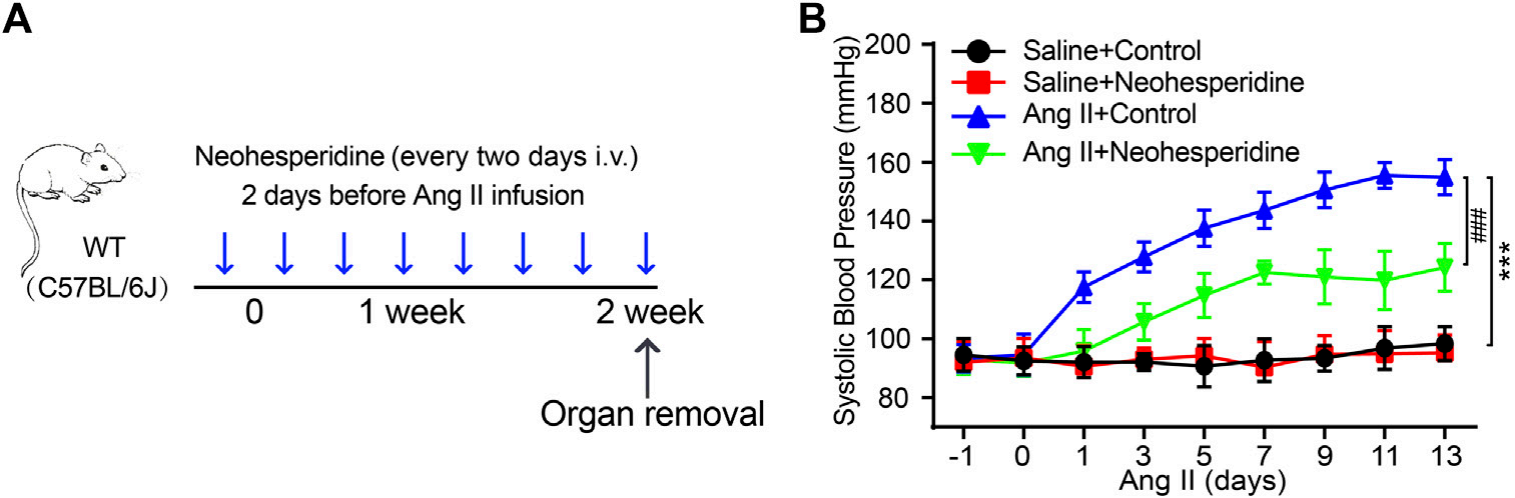
Neohesperidin inhibits hypertension induced by Ang II in mice. Mice were infused with Ang II (490 ng/kg/min) by Alzetmodel 1,002 micropump for 2 weeks. A caudal vein injection of Neohesperidin (50 mg/kg/day) was performed for 16 days **(A)** Schematic diagram of the whole process of the experiment; **(B)** An average systolic blood pressure of each group (*n* = 6). Statistical analysis was performed with one-way ANOVA. ***p < 0.001, sham vs. Ang II; ###*p* < 0.001, Ang II vs. Ang II + Neohesperidin.

We randomly divided the mice into four groups (Sham, Neohesperidin, Ang II and Ang II + Neohesperidin) (*n* = 6). Neohesperidin was injected into the caudal vein 2 days before Ang II infusion (Figure 1A). The blood pressure of mice was measured every 2 days. The schematic diagram is shown in Figure 1A. The result indicated that blood pressure of mice treated with Ang II was significantly higher than that of the control group. Moreover, Neohesperidin treatment statistically ameliorated Ang II induced elevation of blood pressure (Figure 1B). This data indicated that Neohesperidin plays a protective role in Ang II induced hypertension.

### Neohesperidin Attenuates Endothelial Dysfunction Induced by Ang II in Mice

**FIGURE 2:**
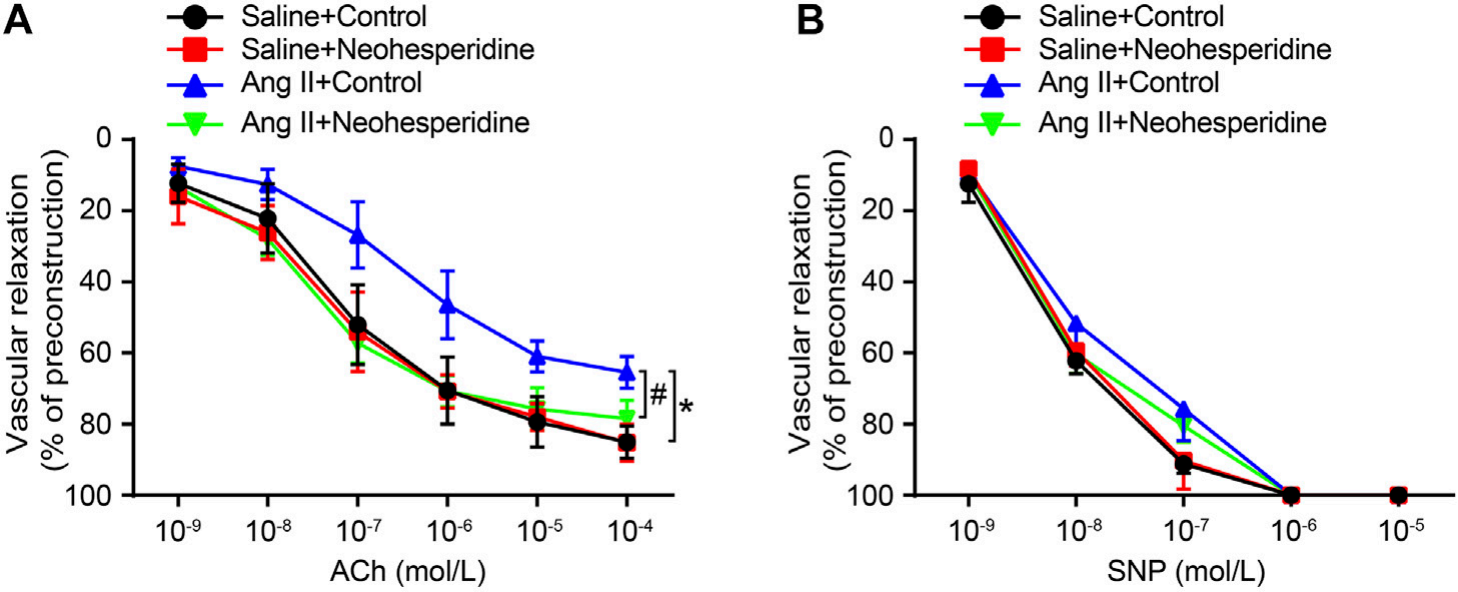
Neohesperidin can prevent endothelial dysfunction induced by Ang II in mice. Mice were infused with Ang II (490 ng/kg/min) by Alzetmodel 1,002 micropump for 2 weeks. A caudal vein injection of Neohesperidin (50 mg/kg/day) was performed for 16 days. Aortic rings were isolated from each group **(A)** We examined endothelium-dependent relaxation in response to different doses of acetylcholine (*n* = 6) **(B)** We examined endothelium-independent relaxation in response to different doses of sodium nitroprusside (*n* = 6). Statistical analysis was performed with one-way ANOVA.*p < 0.05, sham vs. Ang II; #*p* < 0.05, Ang II vs. Ang II + Neohesperidin.

The classicalmethod for detecting vascular endothelial function is vascular relaxation examination. In this paper, we investigated whether Neohesperidin could improve Ang II induced vascular dysfunction. The results showed that Ang II significantly reduced the effect of ACh on endothelium-dependent vasodilation compared with saline control, while Neohesperidin observably significantly preserved vasodilation (Figure 2A). But no significant difference was observed in endothelium-independent vasodilation of SNPs among the four groups (Figure 2B). Therefore, Neohesperidin can protect against Ang II induced endothelial dysfunction.

### Neohesperidin Alleviates Ang II-Induced Vascular Hypertrophy and Sclerosis

**FIGURE 3:**
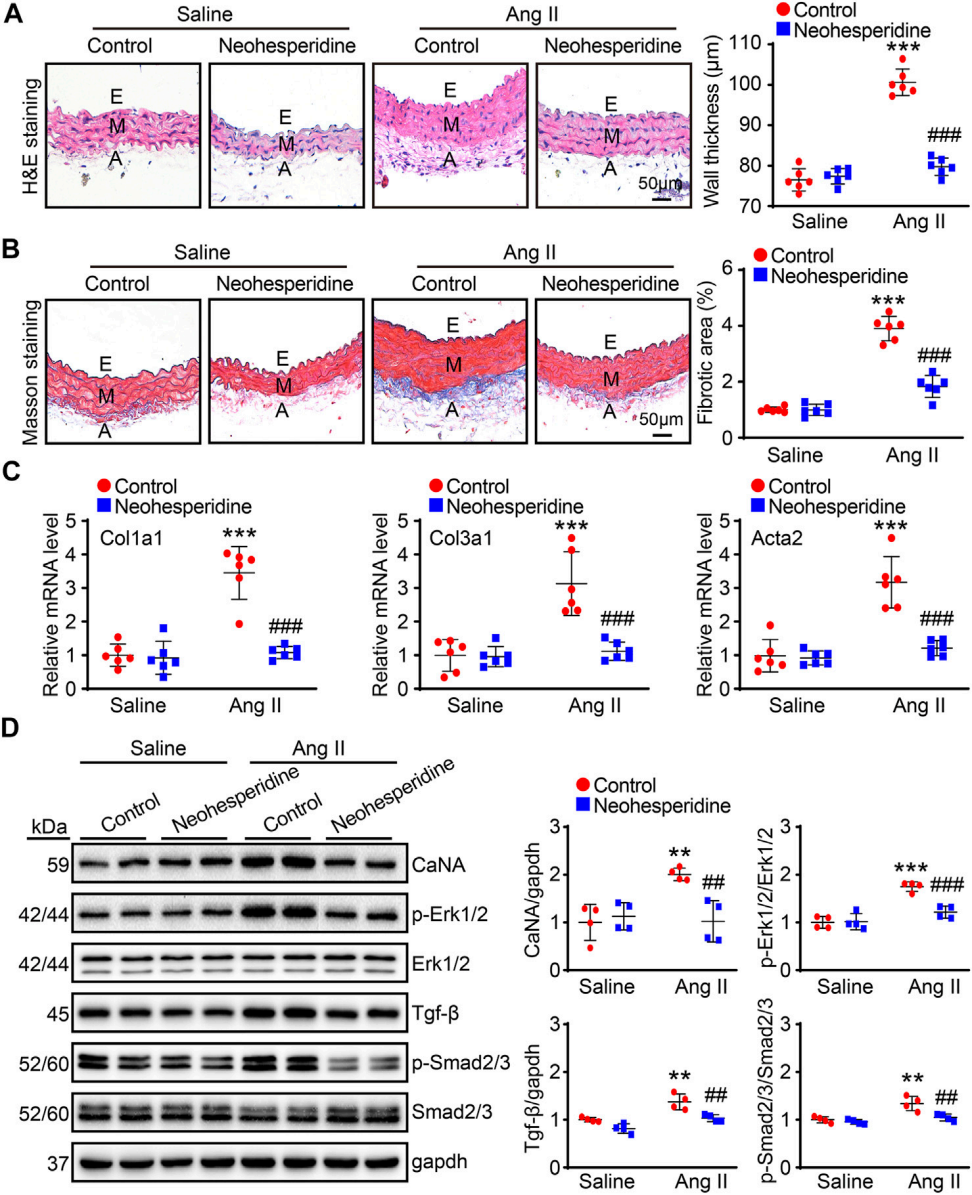
Neohesperidin alleviates Ang II-induced vascular hypertrophy and sclerosis. Mice were infused with Ang II (490 ng/kg/min) by Alzetmodel 1,002 micropump for 2 weeks. A caudal vein injection of Neohesperidin (50 mg/kg/day) was performed for 16 days **(A)** Representative images of H&E staining from each group (left panel, Bar = 50 μm). Histogram of measured aortic wall thickness (right panel, *n* = 6) **(B)** Representative images of Masson’s trichrome staining from each group (left panel, Bar = 50 μm). Histogram of measured fibrotic area (right panel, *n* = 6) **(C)** qPCR analysis of Col1a1, Col3a1, and Acta2 mRNA levels from each group (*n* = 6) **(D)** the protein level of CaNA, p-Erk1/2, Erk1/2, Tgf-β, Smad2/3, and p-Smad2/3 (*n* = 4). Statistical analysis was performed with one-way ANOVA. **p < 0.01 and ***p < 0.001, sham vs. Ang II; ##*p* < 0.005 and ###*p* < 0.001, Ang II vs. Ang II + Neohesperidin.

Vascular hypertrophy and sclerosis are the main pathological basis of cardio-cerebral-renal complications caused by hypertension (Papafaklis et al., 2010). In this paper, the thickness of blood vessels in each group was measured by H&E staining. The results showed that the blood vessel thickness of Ang II group was significantly thickened, while the Ang II group was significantly reduced after Neohesperidin treatment (Figure 3A). Ang II induces vascular dysfunction, tissue fibrosis and remodeling. Similarly, in this paper, we found that after Ang II induction, the degree of vascular fibrosis and the mRNA levels of related factors (Col1a1, Col3a1, and Acta2) in mice were significantly increased, while after Neohesperidin treatment, all indicators were decreased (Figures 3B,C). Next, we examined signaling pathway-related proteins involved in vascular hypertrophy and sclerosis. The results showed that the protein level of CaNA, p-Erk1/2, Tgf-β, and p-Smad2/3 were significantly increased in Ang II group, compared with control group. While these protein levels were decreased after Neohesperidin treatment (Figure 3D).

### Neohesperidin Alleviates Ang II-Induced Vascular Inflammation and Oxidative Stress

**FIGURE 4:**
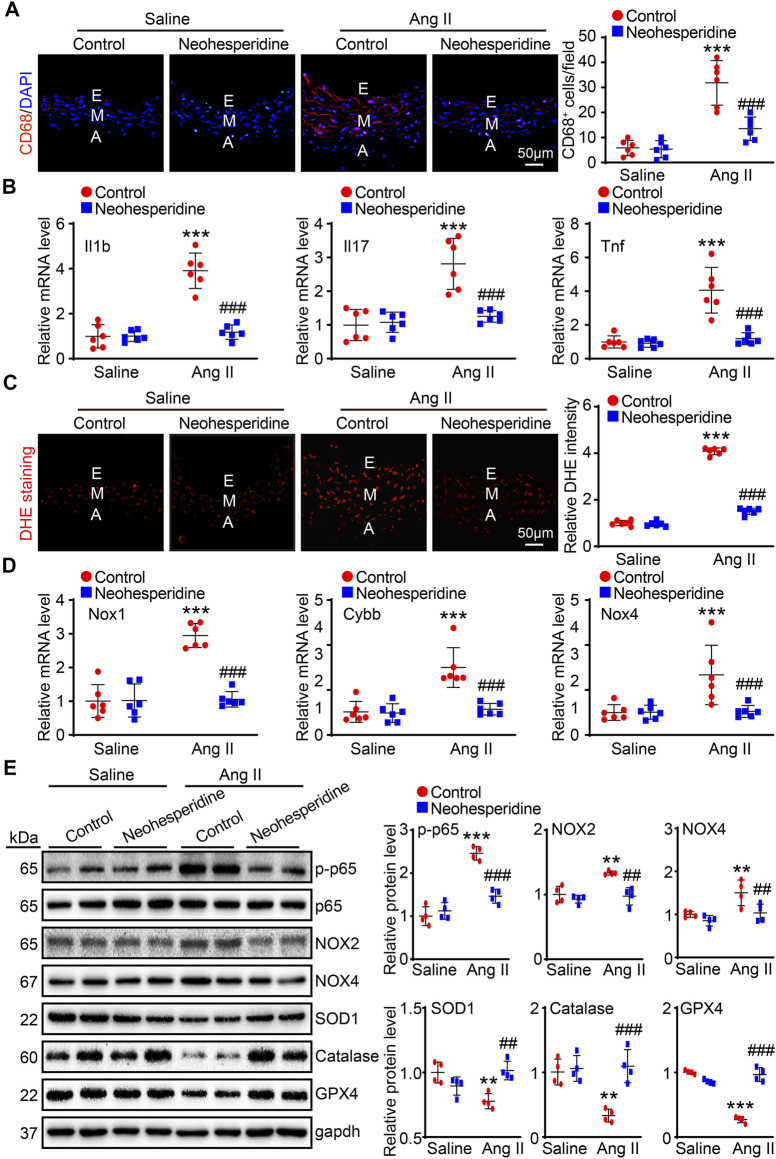
Neohesperidin alleviates Ang II-induced vascular inflammation and oxidative stress. Mice were infused with Ang II (490 ng/kg/min) by Alzetmodel 1,002 micropump for 2 weeks. A caudal vein injection of Neohesperidin (50 mg/kg/day) was performed for 16 days **(A)** Representative images of CD68 staining from each group (left panel, Bar = 50 μm). Histogram of CD68^+^ cells (right panel, *n* = 6); **(B)** qPCR analysis of Il1b, Il17, and Tnf mRNA levels from each group (*n* = 6). **(C)** Representative images of DHE staining from each group (left panel, Bar = 50 μm). Histogram of relative DHE intensity (right panel, *n* = 6); **(D)** qPCR analysis of Nox1, Cybb, and Nox4 mRNA levels from each group (*n* = 6). **(E)** The protein levels of p-p65, p65, NOX2, NOX4, SOD1, Catalase, and GPX4 (*n* = 4). Statistical analysis was performed with one-way ANOVA. **p < 0.01 and ***p < 0.001, sham vs. Ang II; ##*p* < 0.005 and ###*p* < 0.001, Ang II vs. Ang II + Neohesperidin.

Recent studies have found that inflammation and hypertension interact with each other. In this paper, we used CD68 immunofluorescence staining, and the results showed that the aortic infiltration of CD68 positive macrophages and the mRNA expression levels of inflammation-related factors (Il1b, Il17, and Tnf) of Ang II group were significantly enhanced, while these effects were markedly alleviated after Neohesperidin treatment (Figures 4A,B). Endothelial dysfunction contributes to the formation of an inflammatory environment. Similarly, in this paper, the fluorescence intensity of DHE and the mRNA expression levels of related factors (Nox1, Cybb, and Nox4) in blood vessels of the Ang II group were observably increased, while Neohesperidin treatment dramatically inhibited these effects (Figures 4C,D). Next, we examined signaling pathway-related proteins involved in vascular inflammation and oxidative stress. The results showed that the protein level of p-p65, NOX2, and NOX4 were significantly increased in Ang II group, the protein level of SOD1, Catalase and GPX4 were significantly decreased in Ang II group, compared to control group. While Neohesperidin treatment prominently attenuated Ang II-induced abnormal expression of these proteins (Figure 4E).

### Neohesperidin Inhibits Ang II-Induced ROS Production and DNA Damage in HUVECs

**FIGURE 5:**
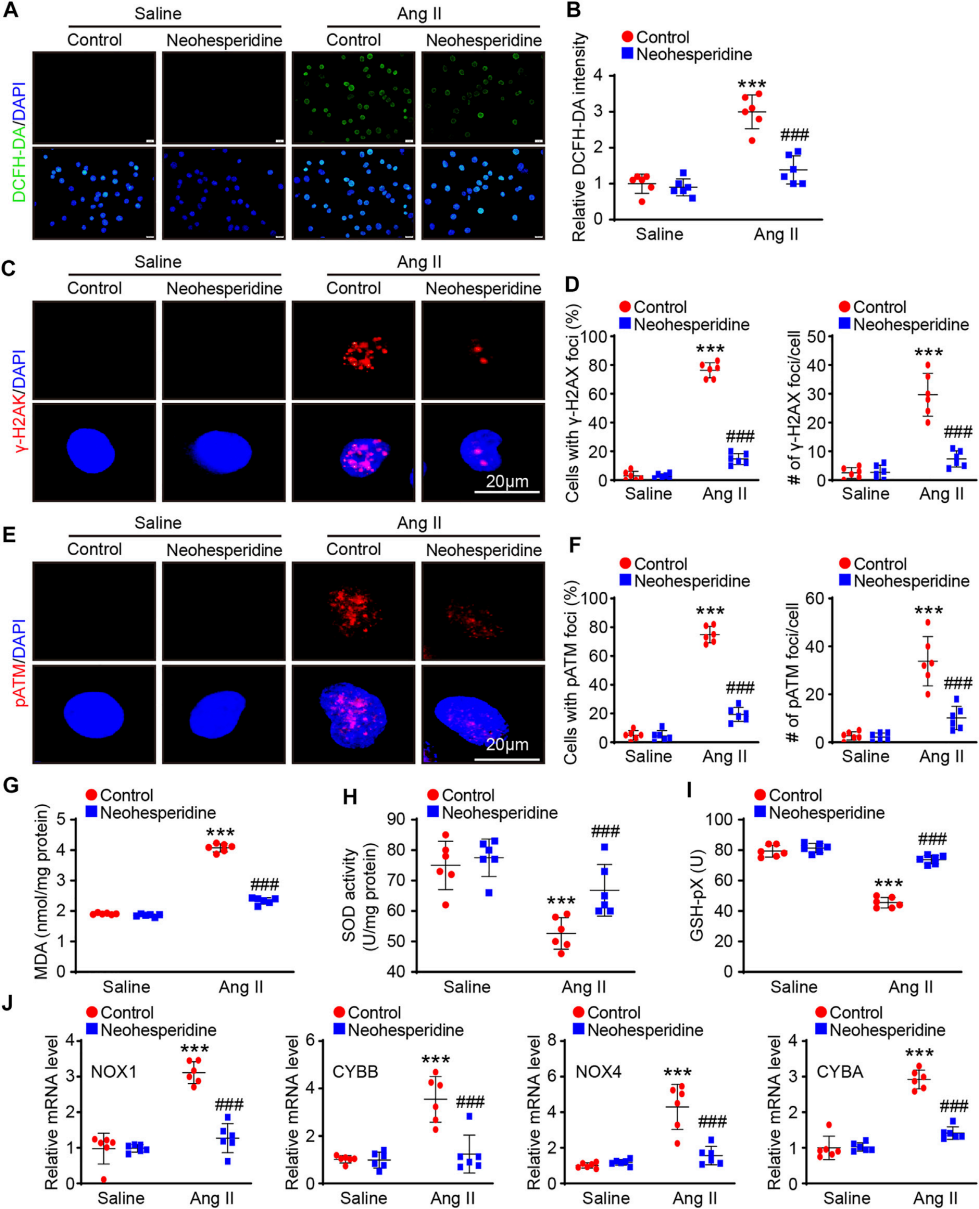
Neohesperidin inhibits Ang II-induced HUVECs ROS and DNA Damage. After cells were treated with Neohesperidin was (20 μm) for 24 h, Ang II (2 μm) were added into cells for 24 h. **(A)** Representative images of DCFH-DA staining from each group (Bar = 20 μm); **(B)** Histogram of the DCFH-DA intensity *n* = 6); **(C)** Representative images of γ-H2AX staining from each group (Bar = 20 μm); **(D)** Histogram of the percentage of HUVCEs with positive γ-H2AX-positive nuclear foci (left, *n* = 6) and the average number of γ-H2AX foci in γ-H2AX-positive HUVECs (right, *n* = 6); **(E)** Representative images of p-ATM staining from each group (Bar = 20 μm); **(F)** Histogram of the percentage of HUVCEs with positive p-ATM -positive nuclear foci (left, *n* = 6) and the average number of p-ATM foci in p-ATM -positive HUVECs (right, *n* = 6); **(G)** Histogram of Malondialdehyde (MDA, *n* = 6); **(H)** Histogram of Superoxide dismutase (SOD, *n* = 6); **(I)** Histogram of Glutathione peroxidase (GSH-Px, *n* = 6); **(J)** qPCR analysis of NOX1, CYBB, NOX4, and CYBA mRNA levels from each group (*n* = 6). Statistical analysis was performed with one-way ANOVA. ***p < 0.001, sham vs. Ang II; ###*p* < 0.001, Ang II vs. Ang II + Neohesperidin.

We have demonstrated that Neohesperidin can inhibit Ang II-induced hypertension in mice and speculate that it may act by attenuating endothelial damage *in vivo*. Next we tested this assumption *in vitro* experiments Vascular endothelial injury is an important indicator of vascular remodeling, which is mainly involved in oxidative stress. We first used DCFH-DA staining to determine whether Neohesperidin regulates oxidative stress in HUVECs. The results showed that the fluorescence intensity of DCFH-DA was increased after Ang II-induced, while the fluorescence intensity of DCFH-DA was decreased after Neohesperidin treatment (Figures 5A,B). H2A Histone Family Member X gamma (γ-H2AX) and p-Ataxia telangiectasia mutated (p-ATM) are markers of cellular oxidative stress and DNA damage. We detected the expression number of γ-H2AX in each group’s cell nucleus. Ang II could significantly promote the expression of γ-H2AX, and also promote the expression of γ-H2AX in each cell nucleus, while the addition of Neohesperidin inhibited this tendency (Figures 5C,D). Ang II could significantly promote the expression of p-ATM and also promote the expression of p-ATM in each cell nucleus, while the addition of Neohesperidin inhibited this tendency (Figures 5E,F). Oxidative stress is closely related to vascular endothelial injury. When MDA increases, the level of oxidation increases. SOD and Glutathione peroxidase (GSH-PX) increase, the level of antioxidant increases. In this paper, we examined the expression levels of these three factors. The results showed that the expression of MDA increased and the expression of SOD and GSH-PX decreased after Ang II stimulation. This is consistent with the literature. Compared with Ang II group, the expression of MDA decreased while the expression of SOD and GSH-PX increased after adding Neohesperidin (Figures 5G–I). Next, the degree of ROS mRNA levels of related factors (NOX1, CYBB, NOX4, and CYBA) in mice were significantly increased, while after Neohesperidin treatment, all indicators were decreased (Figure 5J).

### Neohesperidin Inhibits Ang II-Induced VSMCs Migration

**FIGURE 6:**
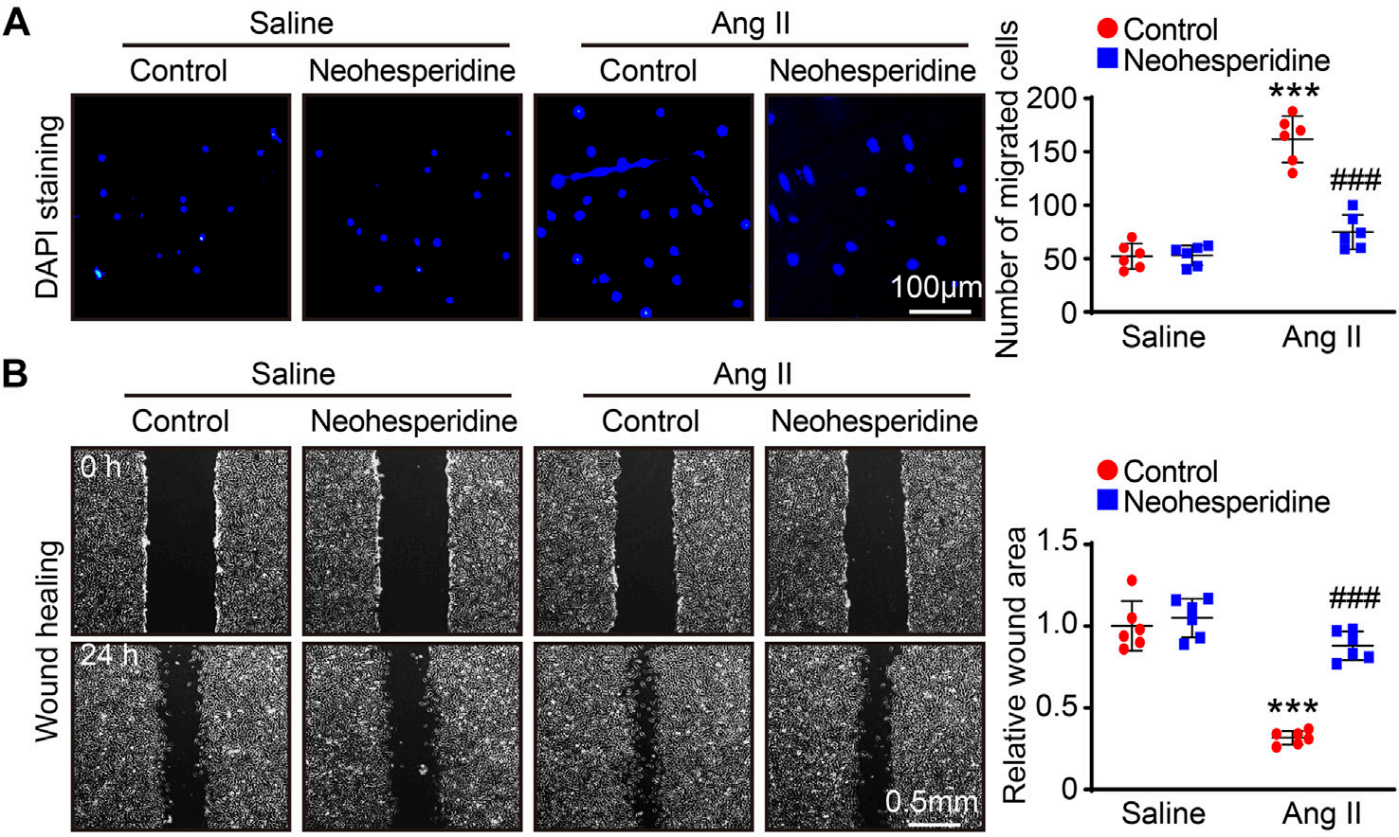
Neohesperidin inhibits Ang II-induced VSMCs migration. After cells were treated with Neohesperidin was (20 μm) for 24 h, Ang II (2 μm) were added into cells for 24 h **(A)** Representative images of DAPI staining from each group and Histogram of the number of VSMCs (*n* = 6) **(B)** Representative images of wound-healing from each group and Histogram of the relative wound area of each group (*n* = 6). Statistical analysis was performed with one-way ANOVA. ***p < 0.001, sham vs. Ang II; ###*p* < 0.001, Ang II vs. Ang II + Neohesperidin.

We then examined the migration ability of VSMCs by transwell and wound-healing assay. The results showed that the migration ability of VSMCs was enhanced by Ang II stimulation. After Neohesperidin was added, the migration ability of VSMCs was weakened (Figures 6A,B).

## Discussion

In recent years, the incidence of cardiovascular disease has been increasing, and it has surpassed cancer as the leading cause of death in Western countries. Hypertension, as a prominent disease in an aging society, is a common chronic disease that can cause myocardial infarction, heart failure, cerebrovascular accident, and other cardiovascular and cerebrovascular risk events. (Madhur et al., 2021). Hypertension not only seriously endangers human health, but also causes a huge economic and social burden. As a multifactorial disease, although the current theories of renin-angiotensin-aldosterone system activation, sympathetic nervous system hyperactivity, and renal water and sodium retention are more recognized, it still cannot fully explain the pathological process of hypertension. It is reported that Ang II is involved in inflammatory response, cell growth, ECM deposition and blood coagulation and other processes, and plays an important role in the process of regulating the structure and function of the blood vessel wall (Ramkumar et al., 2021).

In this study, we established a hypertensive mouse model using Ang II and found thickening of blood vessels, increased blood pressure, and increased markers of oxidative stress and inflammation. Among these complex pathological mechanisms, a common cause is the increased availability of reactive oxygen species, that is, the occurrence of oxidative stress.

Accumulating evidence suggests that oxidative stress and related oxidative damage are the main causes of vascular damage and may be associated with the development of hypertension. ROS is closely related to oxidative stress, and its production is regulated by the antioxidant enzyme system and endothelial nitric oxide synthase. Therefore, how to effectively reduce the occurrence of oxidative stress and the generation of ROS may be a development direction for the treatment of hypertension.

Several pharmacological studies and our lab have found that Neohesperidin can inhibit myocardial hypertrophy and remodeling (Zhang et al., 2020), inhibit allergic reactions (Zhao et al., 2019), reduce pulmonary fibrosis (Zhao et al., 2019), anti-aging (Guo et al., 2019), inhibit tumor occurrence and development, reduce nerve cells apoptosis, improve osteoporosis and so on. Therefore, we hypothesized that Neohesperidin inhibited Ang II-induced vascular remodeling. The results showed that Neohesperidin could indeed inhibit the increase in blood pressure, vessel wall thickening and inflammation induced by Ang II in mice. Studies have shown that Neohesperidin does not cause harm to mice at 50 mg/kg every day, and has the effect of only some diseases. So in this experiment, we also took this therapeutic dose (Lu et al., 2020).

We have demonstrated that Neohesperidin inhibited Ang II-induced vascular remodeling *in vivo*. But blood vessels are structures made up of a variety of cells. We need to further investigate the effect of Neohesperidin on each type of cell. Vascular endothelial cells are an important barrier in the vascular cavity. They not only have the functions of maintaining hemodynamic stability and material exchange, but also secrete inflammatory factors and vasodilation and contraction factors, which play an important role in the regulation of blood pressure. Vascular endothelial dysfunction is one of the important factors that promote the occurrence and development of hypertension. Oxidative stress is an important cause of vascular endothelial damage (Munzel et al., 2017). Because of its ability to participate in angiogenesis, regulate the exchange of macromolecules and inflammatory cells inside and outside the blood vessels, and can produce a variety of active mediators when activated, vascular endothelial cells (VEC) have become the mainstay of cardiovascular, tumor, inflammation, immunity, pharmacology, etc. target of scientific research. As a classic vascular endothelial cell line, the establishment of HUVEC provides an ideal model for VEC-related research (Pastore et al., 1999; Cai et al., 2019; Yu et al., 2021). In this article, we found that Ang II could cause DNA damage and oxidative stress damage to HUVECs, while Neohesperidin could inhibit Ang II-induced oxidative stress damage *in vitro* experiments. The above experiments confirmed that Neohesperidin has a protective effect on endothelial cells by regulating the changes of oxidative stress.

Vascular remodeling is the key pathological basis of hypertension and target organ injury. Abnormal proliferation and migration of VSMC are central to vascular remodeling. Ang II promotes the formation and development of vascular remodeling in hypertension by promoting proliferation and migration of VSMC (Zou et al., 2022). ROS is involved in smooth muscle cell migration, the expression of inflammatory mediators and matrix component. In this study, we found that Neohesperidin can inhibit Ang II-induced VSMC migration. The result was in line with our expectations.

Although we found Neohesperidin protected against Ang II-induced hypertension and vascular remodeling. However, there is no specific study on which protein or pathway neohesperidin affects the proliferation and migration of vascular smooth muscle and endothelial injury. In the coming work, we would find this phenomenon in proteomics or transcriptomics.

**FIGURE 7:**
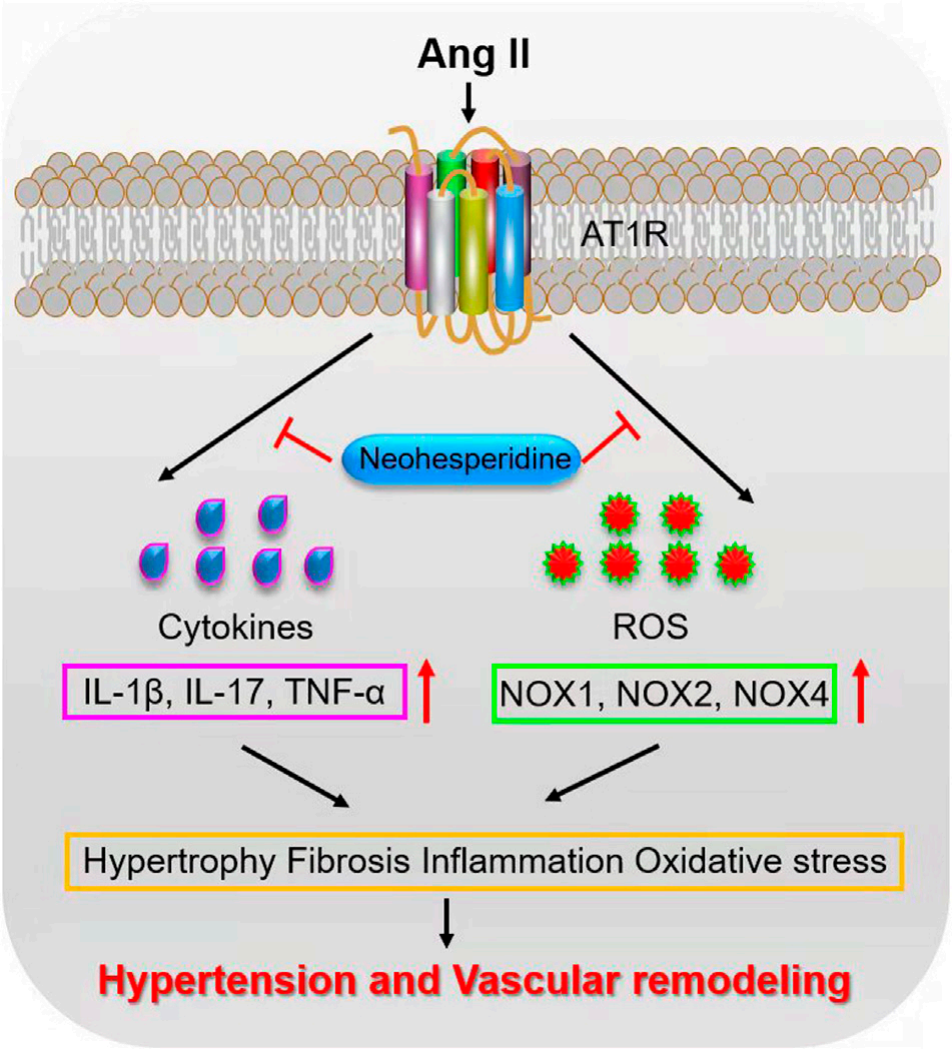
Schematic diagram of the mechanism.

In summary, Neohesperidin could modulate Ang II-induced vascular remodeling in mice (Figure 7). This finding suggests that Neohesperidin may be a potential target for the development of anti-hypertensive drugs.

## Data Availability

The raw data supporting the conclusions of this article will be made available by the authors, without undue reservation.
