# Supplementary material for: Neohesperidin Protects Angiotensin II-Induced Hypertension and Vascular Remodeling
Source: Front Pharmacol. 2022 May 23;13:890202. doi: 10.3389/fphar.2022.890202 (PMC9168427; doi:10.3389/fphar.2022.890202)
Supplement: Supplementary file 1 [file DataSheet1.docx]

**Raw data presented in our tables and figures**

**Figure 1A**

**Figure 1B**

Blood pressure

| Saline + Control | | | | | | |
| --- | --- | --- | --- | --- | --- | --- |
|  | 1# | 2# | 3# | 4# | 5# | 6# |
| C1 | 88 | 95 | 103 | 98 | 89 | 94 |
| C2 | 88 | 95 | 93 | 100 | 92 | 87 |
| A1 | 91 | 102 | 88 | 87 | 91 | 93 |
| A3 | 90 | 95 | 88 | 91 | 93 | 95 |
| A5 | 82 | 91 | 87 | 93 | 88 | 103 |
| A7 | 98 | 87 | 104 | 84 | 91 | 92 |
| A9 | 100 | 88 | 94 | 89 | 94 | 95 |
| A11 | 102 | 96 | 83 | 102 | 101 | 97 |
| A13 | 98 | 105 | 97 | 89 | 97 | 104 |
| Saline + Neohesperidin | | | | | | |
|  | 1# | 2# | 3# | 4# | 5# | 6# |
| C1 | 89 | 99 | 102 | 91 | 84 | 87 |
| C2 | 85 | 96 | 98 | 102 | 93 | 87 |
| A1 | 96 | 84 | 101 | 92 | 84 | 87 |
| A3 | 93 | 100 | 89 | 91 | 94 | 91 |
| A5 | 87 | 94 | 102 | 93 | 89 | 100 |
| A7 | 103 | 92 | 96 | 84 | 88 | 79 |
| A9 | 96 | 100 | 85 | 97 | 89 | 101 |
| A11 | 97 | 81 | 101 | 103 | 92 | 95 |
| A13 | 100 | 97 | 103 | 86 | 93 | 92 |
| Ang II | | | | | | |
|  | 1# | 2# | 3# | 4# | 5# | 6# |
| C1 | 101 | 95 | 88 | 90 | 91 | 95 |
| A1 | 94 | 88 | 86 | 94 | 102 | 103 |
| C2 | 125 | 116 | 118 | 120 | 109 | 117 |
| A3 | 134 | 123 | 121 | 132 | 127 | 130 |
| A5 | 144 | 146 | 135 | 136 | 130 | 134 |
| A7 | 152 | 150 | 141 | 144 | 137 | 138 |
| A9 | 157 | 156 | 146 | 155 | 146 | 143 |
| A11 | 160 | 156 | 153 | 161 | 150 | 153 |
| A13 | 159 | 163 | 152 | 158 | 149 | 148 |
| Ang II + Neohesperidin | | | | | | |
|  | 1# | 2# | 3# | 4# | 5# | 6# |
| C1 | 89 | 93 | 101 | 86 | 94 | 95 |
| A1 | 100 | 89 | 93 | 91 | 89 | 88 |
| C2 | 90 | 102 | 87 | 93 | 97 | 106 |
| A3 | 108 | 107 | 94 | 105 | 108 | 112 |
| A5 | 120 | 100 | 117 | 119 | 114 | 118 |
| A7 | 123 | 116 | 120 | 127 | 124 | 125 |
| A9 | 120 | 124 | 124 | 103 | 127 | 128 |
| A11 | 132 | 106 | 127 | 124 | 120 | 110 |
| A13 | 133 | 125 | 128 | 127 | 123 | 109 |

**Figure 2A**

**ACH**

Saline + Control

|  | ACH-9 | ACH-8 | ACH-7 | ACH-6 | ACH-5 | ACH-4 |
| --- | --- | --- | --- | --- | --- | --- |
| 1# | 8.47 | 16.95 | 45.76 | 72.88 | 86.44 | 91.53 |
| 2# | 18.18 | 33.33 | 48.48 | 75.76 | 78.79 | 87.88 |
| 3# | 10.53 | 28.95 | 65.79 | 78.95 | 86.84 | 86.84 |
| 4# | 16.92 | 29.23 | 64.62 | 76.92 | 81.54 | 81.54 |
| 5# | 15.00 | 10.00 | 37.00 | 55.00 | 72.00 | 80.00 |
| 6# | 4.71 | 14.12 | 50.59 | 63.53 | 70.59 | 82.35 |

Saline + Neohesperidin

|  | ACH-9 | ACH-8 | ACH-7 | ACH-6 | ACH-5 | ACH-4 |
| --- | --- | --- | --- | --- | --- | --- |
| 1# | 10.00 | 20.00 | 63.33 | 73.33 | 76.67 | 83.33 |
| 2# | 18.18 | 22.73 | 63.64 | 72.73 | 77.27 | 81.82 |
| 3# | 24.00 | 40.00 | 64.00 | 76.00 | 84.00 | 92.00 |
| 4# | 3.64 | 20.00 | 49.09 | 70.91 | 72.73 | 78.18 |
| 5# | 20.00 | 27.27 | 45.45 | 69.09 | 78.18 | 89.09 |
| 6# | 20.00 | 26.67 | 38.67 | 62.67 | 78.67 | 86.67 |

Ang II + Control

|  | ACH-9 | ACH-8 | ACH-7 | ACH-6 | ACH-5 | ACH-4 |
| --- | --- | --- | --- | --- | --- | --- |
| 1# | 7.27 | 20.00 | 25.45 | 55.45 | 62.73 | 62.73 |
| 2# | 6.15 | 11.46 | 33.85 | 50.77 | 65.38 | 66.92 |
| 3# | 10.43 | 13.04 | 38.26 | 44.35 | 63.04 | 69.13 |
| 4# | 4.04 | 8.08 | 17.17 | 56.57 | 62.63 | 69.70 |
| 5# | 7.62 | 9.52 | 15.24 | 33.33 | 58.10 | 58.10 |
| 6# | 9.23 | 13.46 | 30.77 | 38.46 | 53.85 | 66.15 |

Ang II + Neohesperidin

|  | ACH-9 | ACH-8 | ACH-7 | ACH-6 | ACH-5 | ACH-4 |
| --- | --- | --- | --- | --- | --- | --- |
| 1# | 10.59 | 20.00 | 54.12 | 67.06 | 72.94 | 76.47 |
| 2# | 15.38 | 30.77 | 61.54 | 66.92 | 70.00 | 75.31 |
| 3# | 13.33 | 26.67 | 66.67 | 77.78 | 77.22 | 78.89 |
| 4# | 21.05 | 29.47 | 53.68 | 73.68 | 83.16 | 83.68 |
| 5# | 10.20 | 27.06 | 54.12 | 69.41 | 69.35 | 71.76 |
| 6# | 9.20 | 33.33 | 53.33 | 69.33 | 81.33 | 84.67 |

**Figure 2B**

**SNP**

Saline + Control

|  | SNP-9 | SNP-8 | SNP-7 | SNP-6 | SNP-5 |
| --- | --- | --- | --- | --- | --- |
| 1# | 11.06 | 61.18 | 88.00 | 100.00 | 100.00 |
| 2# | 6.00 | 64.00 | 94.00 | 100.00 | 100.00 |
| 3# | 15.78 | 59.00 | 91.00 | 100.00 | 100.00 |
| 4# | 21.05 | 66.00 | 88.00 | 100.00 | 100.00 |
| 5# | 10.67 | 57.00 | 94.00 | 100.00 | 100.00 |
| 6# | 10.09 | 66.00 | 92.00 | 100.00 | 100.00 |

Saline + Neohesperidin

|  | SNP-9 | SNP-8 | SNP-7 | SNP-6 | SNP-5 |
| --- | --- | --- | --- | --- | --- |
| 1# | 6.00 | 65.00 | 78.00 | 100.00 | 100.00 |
| 2# | 12.00 | 64.00 | 84.00 | 100.00 | 100.00 |
| 3# | 10.00 | 57.00 | 98.00 | 100.00 | 100.00 |
| 4# | 7.00 | 60.00 | 90.00 | 100.00 | 100.00 |
| 5# | 10.45 | 56.00 | 94.00 | 100.00 | 100.00 |
| 6# | 4.00 | 55.00 | 98.00 | 100.00 | 100.00 |

Ang II + Control

|  | SNP-9 | SNP-8 | SNP-7 | SNP-6 | SNP-5 |
| --- | --- | --- | --- | --- | --- |
| 1# | 9.08 | 42.00 | 65.00 | 100.00 | 100.00 |
| 2# | 10.26 | 44.00 | 66.00 | 100.00 | 100.00 |
| 3# | 9.48 | 52.00 | 73.00 | 100.00 | 100.00 |
| 4# | 10.49 | 58.00 | 85.00 | 100.00 | 100.00 |
| 5# | 9.55 | 58.00 | 85.00 | 100.00 | 100.00 |
| 6# | 7.07 | 56.00 | 80.00 | 100.00 | 100.00 |

Ang II + Neohesperidin

|  | SNP-9 | SNP-8 | SNP-7 | SNP-6 | SNP-5 |
| --- | --- | --- | --- | --- | --- |
| 1# | 10.67 | 53.33 | 85.00 | 100.00 | 100.00 |
| 2# | 9.23 | 67.69 | 76.00 | 100.00 | 100.00 |
| 3# | 16.36 | 56.36 | 84.00 | 100.00 | 100.00 |
| 4# | 3.85 | 60.00 | 85.00 | 100.00 | 100.00 |
| 5# | 8.00 | 66.00 | 76.00 | 100.00 | 100.00 |
| 6# | 8.70 | 58.26 | 77.00 | 100.00 | 100.00 |

**Figure 3A**

H&E staining of the aorta

2) Quantification of wall thickness

| Saline + Control | | | | | |
| --- | --- | --- | --- | --- | --- |
| 76 | 81 | 75 | 73 | 78 | 76 |
| Saline + Neohesperidin | | | | | |
| 74.18 | 79.32 | 77.67 | 76.53 | 79.12 | 77.45 |
| Ang II | | | | | |
| 98.55 | 106.43 | 102.15 | 100 | 97.25 | 99.37 |
| Ang II + Neohesperidin | | | | | |
| 76.45 | 80.12 | 78.53 | 81.47 | 82.54 | 79.47 |

**Figure 3B**

1) Masson staining of the aorta

2) Quantification of fibrotic area

| Saline + Control | | | | | |
| --- | --- | --- | --- | --- | --- |
| 1.055 | 0.976 | 0.909 | 0.946 | 0.951 | 1.162 |
| Saline + Neohesperidin | | | | | |
| 1.27 | 0.95 | 0.88 | 0.715 | 1.192 | 0.962 |
| Ang II | | | | | |
| 4.51 | 3.496 | 3.305 | 3.978 | 4.04 | 4.09 |
| Ang II + Neohesperidin | | | | | |
| 2.359 | 1.97 | 1.783 | 1.735 | 1.972 | 1.164 |

**Figure 3C**

qPCR analysis of collagen I, collagen III and α-SMA mRNA levels

|  | gapdh | Collagen I | Normalization | Collagen III | Normalization | a-SMA | Normalization |
| --- | --- | --- | --- | --- | --- | --- | --- |
| Saline + Control | 18.12 | 28.11 | 0.97 | 27.12 | 1.09 | 24.23 | 1.11 |
|  | 19.21 | 28.91 | 1.19 | 27.85 | 1.39 | 24.56 | 1.89 |
|  | 18.56 | 28.76 | 0.84 | 27.36 | 1.25 | 25.31 | 0.71 |
|  | 18.55 | 27.88 | 1.54 | 27.15 | 1.43 | 25.16 | 0.79 |
|  | 18.26 | 28.34 | 0.91 | 28.45 | 0.48 | 24.67 | 0.90 |
|  | 17.68 | 28.46 | 0.56 | 28.34 | 0.34 | 24.88 | 0.52 |
| Saline + Neohesperidin | 17.28 | 28.76 | 0.35 | 26.66 | 0.83 | 24.033 | 0.71 |
|  | 17.93 | 28.11 | 0.85 | 26.45 | 1.51 | 24.14 | 1.04 |
|  | 18.05 | 28.56 | 0.68 | 27.55 | 0.77 | 24.03 | 1.22 |
|  | 18.03 | 28.33 | 0.79 | 27.16 | 0.99 | 24.69 | 0.76 |
|  | 18.66 | 27.76 | 1.80 | 28.39 | 0.65 | 24.88 | 1.03 |
|  | 17.98 | 27.83 | 1.07 | 27.11 | 0.99 | 24.67 | 0.75 |
| Ang II | 17.56 | 26.56 | 1.93 | 25.11 | 2.96 | 22.12 | 3.26 |
|  | 17.82 | 25.89 | 3.68 | 25.58 | 2.56 | 22.45 | 3.11 |
|  | 18.08 | 26.31 | 3.30 | 25.99 | 2.31 | 22.61 | 3.33 |
|  | 18.91 | 26.89 | 3.92 | 25.86 | 4.49 | 23.01 | 4.49 |
|  | 18.66 | 26.67 | 3.84 | 25.73 | 4.13 | 23.66 | 2.40 |
|  | 18.44 | 26.38 | 4.03 | 26.34 | 2.33 | 23.43 | 2.42 |
| Ang II + Neohesperidin | 17.77 | 27.52 | 1.15 | 27.01 | 0.92 | 24.08 | 0.97 |
|  | 17.64 | 27.86 | 0.83 | 26.58 | 1.13 | 23.35 | 1.47 |
|  | 17.38 | 27.08 | 1.19 | 26.05 | 1.36 | 23.11 | 1.45 |
|  | 18.11 | 27.64 | 1.34 | 27.31 | 0.94 | 24.06 | 1.24 |
|  | 18.09 | 28.04 | 1.00 | 27.46 | 0.84 | 24.11 | 1.19 |
|  | 18.05 | 28.06 | 0.96 | 26.55 | 1.53 | 24.37 | 0.96 |

**Figure 4A**

1) CD68 staining

2) analysis of the CD68-positive area

| Saline + Control | | | | | |
| --- | --- | --- | --- | --- | --- |
| 3 | 8 | 5 | 7 | 10 | 2 |
| Saline + Neohesperidin | | | | | |
| 2 | 1 | 5 | 6 | 8 | 10 |
| Ang II + Control | | | | | |
| 22 | 38 | 20 | 33 | 42 | 36 |
| Ang II + Neohesperidin | | | | | |
| 12 | 8 | 9 | 20 | 15 | 17 |

**Figure 4B:** qPCR analysis of the IL-1β, IL-17 and TNF-α mRNA levels in the aorta

|  | gapdh | IL-1β | Normalization | IL-17 | Normalization | TNF-a | Normalization |
| --- | --- | --- | --- | --- | --- | --- | --- |
| Saline + Control | 18.12 | 29.56 | 1.00 | 26.66 | 0.61 | 30.25 | 0.97 |
|  | 19.21 | 29.86 | 1.73 | 26.45 | 1.50 | 30.56 | 1.67 |
|  | 18.56 | 29.45 | 1.46 | 25.98 | 1.33 | 30.75 | 0.93 |
|  | 18.55 | 30.25 | 0.83 | 25.88 | 1.41 | 30.66 | 0.98 |
|  | 18.26 | 30.87 | 0.44 | 26.73 | 0.64 | 30.91 | 0.68 |
|  | 17.68 | 30.11 | 0.50 | 26.54 | 0.49 | 30.28 | 0.70 |
| Saline + Neohesperidin | 17.28 | 29.22 | 0.71 | 25.87 | 0.59 | 30.02 | 0.64 |
|  | 17.93 | 29.45 | 0.95 | 25.44 | 1.25 | 30.22 | 0.87 |
|  | 18.05 | 29.61 | 0.92 | 25.39 | 1.40 | 30.09 | 1.03 |
|  | 18.03 | 29.08 | 1.31 | 26.01 | 0.90 | 29.88 | 1.18 |
|  | 18.66 | 29.76 | 1.27 | 26.11 | 1.30 | 30.56 | 1.14 |
|  | 17.98 | 29.67 | 0.84 | 25.77 | 1.03 | 30.72 | 0.64 |
| Ang II + Control | 17.56 | 27.56 | 2.71 | 24.35 | 2.05 | 28.46 | 2.28 |
|  | 17.82 | 27.35 | 3.76 | 23.79 | 3.63 | 28.16 | 3.36 |
|  | 18.08 | 27.46 | 4.17 | 24.11 | 3.48 | 28.37 | 3.47 |
|  | 18.91 | 28.01 | 5.06 | 25.02 | 3.29 | 28.76 | 4.71 |
|  | 18.66 | 28.02 | 4.23 | 25.16 | 2.51 | 28.64 | 4.31 |
|  | 18.44 | 28.06 | 3.53 | 25.34 | 1.90 | 27.89 | 6.22 |
| Ang II + Neohesperidin | 17.77 | 29.13 | 1.06 | 25.56 | 1.03 | 29.37 | 1.40 |
|  | 17.64 | 28.88 | 1.15 | 24.95 | 1.43 | 29.88 | 0.90 |
|  | 17.38 | 28.67 | 1.11 | 24.98 | 1.17 | 29.64 | 0.89 |
|  | 18.11 | 28.85 | 1.62 | 25.76 | 1.13 | 29.35 | 1.80 |
|  | 18.09 | 30.07 | 0.69 | 25.53 | 1.31 | 30.11 | 1.05 |
|  | 18.05 | 28.97 | 1.43 | 25.33 | 1.46 | 30.05 | 1.06 |

**Figure 4C**

1) DHE fluorescence staining of the aorta

2) quantification of relative fluorescence intensity

| Saline + Control | | | | | |
| --- | --- | --- | --- | --- | --- |
| 1.149 | 0.942 | 0.839 | 1.11 | 0.96 | 1.001 |
| Saline + Neohesperidin | | | | | |
| 1.153 | 0.91 | 0.846 | 0.894 | 0.982 | 0.968 |
| Ang II + Control | | | | | |
| 3.828 | 4.183 | 4.206 | 4.17 | 4.119 | 3.991 |
| Ang II + Neohesperidin | | | | | |
| 1.6166 | 1.2643 | 1.5982 | 1.518 | 1.5985 | 1.4568 |

**Figure 4D:** qPCR analysis of the NOX1, NOX2 and NOX4 mRNA levels

|  | gapdh | NOX1 | Normalization | NOX2 | Normalization | NOX4 | Normalization |
| --- | --- | --- | --- | --- | --- | --- | --- |
| Saline + Control | 18.12 | 29.84 | 0.85 | 27.68 | 0.78 | 28.65 | 0.68 |
|  | 19.21 | 29.78 | 1.88 | 27.48 | 1.91 | 28.46 | 1.64 |
|  | 18.56 | 29.76 | 1.21 | 27.55 | 1.16 | 28.55 | 0.98 |
|  | 18.55 | 30.21 | 0.88 | 27.91 | 0.90 | 28.37 | 1.11 |
|  | 18.26 | 30.22 | 0.72 | 28.01 | 0.68 | 28.59 | 0.78 |
|  | 17.68 | 30.17 | 0.50 | 27.33 | 0.73 | 27.99 | 0.79 |
| Saline + Neohesperidin | 17.28 | 29.88 | 0.46 | 27.31 | 0.56 | 27.89 | 0.64 |
|  | 17.93 | 29.99 | 0.67 | 27.09 | 1.03 | 27.63 | 1.20 |
|  | 18.05 | 29.76 | 0.85 | 27.05 | 1.15 | 28.06 | 0.97 |
|  | 18.03 | 29.65 | 0.91 | 27.36 | 0.91 | 28.46 | 0.72 |
|  | 18.66 | 29.43 | 1.64 | 27.14 | 1.65 | 28.06 | 1.48 |
|  | 17.98 | 28.77 | 1.61 | 28.03 | 0.55 | 27.88 | 1.05 |
| Ang II + Control | 17.56 | 27.31 | 3.32 | 25.37 | 2.62 | 26.76 | 1.70 |
|  | 17.82 | 27.65 | 3.14 | 25.68 | 2.53 | 26.66 | 2.18 |
|  | 18.08 | 27.82 | 3.34 | 25.94 | 2.53 | 27.61 | 1.35 |
|  | 18.91 | 28.98 | 2.66 | 26.76 | 2.55 | 26.55 | 5.01 |
|  | 18.66 | 28.73 | 2.66 | 25.61 | 4.76 | 26.93 | 3.24 |
|  | 18.44 | 28.56 | 2.57 | 26.05 | 3.01 | 27.06 | 2.54 |
| Ang II + Neohesperidin | 17.77 | 29.15 | 1.07 | 27.08 | 0.93 | 27.67 | 1.05 |
|  | 17.64 | 29.34 | 0.86 | 26.98 | 0.91 | 27.88 | 0.83 |
|  | 17.38 | 28.88 | 0.99 | 25.99 | 1.51 | 27.66 | 0.80 |
|  | 18.11 | 29.64 | 0.97 | 27.38 | 0.95 | 27.45 | 1.54 |
|  | 18.09 | 28.99 | 1.50 | 27.06 | 1.17 | 27.98 | 1.05 |
|  | 18.05 | 29.61 | 0.95 | 26.77 | 1.39 | 27.99 | 1.02 |

**Figure 5A:** γ-H2AX staining

**Figure 5B**

Histogram of the percentage of HUVCEs with positive γ-H2AX-positive nuclear foci

| Saline + Control | | | | | |
| --- | --- | --- | --- | --- | --- |
| 0 | 1 | 5 | 0 | 8 | 3 |
| Saline + Neohesperidin | | | | | |
| 0 | 1 | 4 | 5 | 3 | 2 |
| Ang II + Control | | | | | |
| 78 | 70 | 80 | 83 | 77 | 70 |
| Ang II + Neohesperidin | | | | | |
| 12 | 13 | 10 | 15 | 20 | 18 |

| Saline + Control | | | | | |
| --- | --- | --- | --- | --- | --- |
| 4 | 1 | 2 | 3 | 0 | 5 |
| Saline + Neohesperidin | | | | | |
| 0 | 5 | 6 | 3 | 0 | 2 |
| Ang II + Control | | | | | |
| 28 | 36 | 20 | 30 | 24 | 40 |
| Ang II + Neohesperidin | | | | | |
| 8 | 11 | 5 | 10 | 4 | 6 |

**Figure 5C:** p-ATM staining

**Figure 5D:** Histogram of the percentage of HUVCEs with positive p-ATM -positive nuclear foci (left) and the average number of p-ATM foci in p-ATM -positive HUVECs

| Saline + Control | | | | | |
| --- | --- | --- | --- | --- | --- |
| 0 | 3 | 5 | 6 | 10 | 5 |
| Saline + Neohesperidin | | | | | |
| 0 | 11 | 2 | 3 | 5 | 5 |
| Ang II + Control | | | | | |
| 68 | 81 | 73 | 70 | 82 | 75 |
| Ang II + Neohesperidin | | | | | |
| 22 | 20 | 18 | 16 | 13 | 27 |

| Saline + Control | | | | | |
| --- | --- | --- | --- | --- | --- |
| 0 | 4 | 2 | 3 | 2 | 5 |
| Saline + Neohesperidin | | | | | |
| 0 | 1 | 1 | 4 | 4 | 3 |
| Ang II + Control | | | | | |
| 20 | 40 | 50 | 33 | 28 | 32 |
| Ang II + Neohesperidin | | | | | |
| 11 | 5 | 6 | 8 | 13 | 18 |

**Figure 5E**

Analysis of the malonaldehyde concentration

| Saline + Control | | | | | |
| --- | --- | --- | --- | --- | --- |
| 1.95 | 1.93 | 1.88 | 1.91 | 1.9 | 1.89 |
| Saline + Neohesperidin | | | | | |
| 1.84 | 1.88 | 1.86 | 1.91 | 1.88 | 1.79 |
| Ang II + Control | | | | | |
| 4.23 | 4.12 | 4.18 | 4.09 | 3.98 | 3.87 |
| Ang II + Neohesperidin | | | | | |
| 2.43 | 2.4 | 2.15 | 2.28 | 2.34 | 2.4 |

**Figure 5F**

Analysis of the SOD levels

| Saline + Control | | | | | |
| --- | --- | --- | --- | --- | --- |
| 80 | 78 | 72 | 85 | 62 | 73 |
| Saline + Neohesperidin | | | | | |
| 82 | 66 | 77 | 83 | 77 | 80 |
| Ang II + Control | | | | | |
| 54 | 46 | 50 | 58 | 49 | 59 |
| Ang II + Neohesperidin | | | | | |
| 60 | 73 | 60 | 81 | 65 | 62 |

**Figure 5G**

Analysis of the GSH levels

| Saline + Control | | | | | |
| --- | --- | --- | --- | --- | --- |
| 84 | 76 | 79 | 84 | 75 | 78 |
| Saline + Neohesperidin | | | | | |
| 81 | 85 | 79 | 82 | 84 | 77 |
| Ang II + Control | | | | | |
| 42 | 50 | 42 | 49 | 46 | 44 |
| Ang II + Neohesperidin | | | | | |
| 74 | 76 | 77 | 75 | 71 | 70 |

**Figure 6A**

DAPI staining of migrated cells

| Saline + Control | | | | | |
| --- | --- | --- | --- | --- | --- |
| 42 | 60 | 55 | 38 | 48 | 70 |
| Saline + Neohesperidin | | | | | |
| 55 | 40 | 43 | 62 | 58 | 60 |
| Ang II + Control | | | | | |
| 188 | 176 | 130 | 165 | 142 | 170 |
| Ang II + Neohesperidin | | | | | |
| 100 | 87 | 60 | 59 | 69 | 74 |

**Figure 6B**

wound-healing

| Saline + Control | | | | | |
| --- | --- | --- | --- | --- | --- |
| 0.94 | 0.9 | 1.05 | 1.28 | 0.86 | 0.98 |
| Saline + Neohesperidin | | | | | |
| 1.11 | 1.16 | 0.89 | 1.17 | 1.04 | 0.93 |
| Ang II + Control | | | | | |
| 0.34 | 0.31 | 0.28 | 0.26 | 0.37 | 0.34 |
| Ang II + Neohesperidin | | | | | |
| 0.83 | 0.97 | 0.92 | 0.77 | 0.81 | 0.98 |
